# Adherence to Cancer Survivorship Care Guidelines and Health Care Utilization Patterns Among Nonmetastatic Breast Cancer Survivors in Singapore

**DOI:** 10.1200/GO.21.00246

**Published:** 2022-04-04

**Authors:** Yu Ke, Chia Jie Tan, Hui Ling Angie Yeo, Alexandre Chan

**Affiliations:** ^1^ Department of Pharmacy, National University of Singapore, Singapore, Singapore; ^2^ Department of Pharmacy, National Cancer Centre Singapore, Singapore; ^3^ Department of Clinical Pharmacy Practice, University of California Irvine, Irvine, CA

## Abstract

Currently, limited information is available on care provided to breast cancer survivors in Singapore. This study aims to assess the quality of post-treatment cancer survivorship care among breast cancer survivors on the basis of compliance with international guidelines up to 5 years post-primary treatment.

**METHODS:**

This study analyzed a cohort of 189 nonmetastatic breast cancer survivors recruited from the National Cancer Centre Singapore, Changi General Hospital, and KK Women's and Children's Hospital between November 2011 and September 2015. Data were retrieved from electronic medical records in 6-month intervals. Adherence to guidelines was assessed in four areas: (1) recurrent cancer surveillance, (2) monitoring and detecting late effects, (3) health care resource utilization, and (4) preventive care. Descriptive statistics, Kaplan-Meier, and regression analyses were conducted.

**RESULTS:**

Annual surveillance mammogram adherence rates were ≥ 83% consistently. The most common new diagnosis was osteoporosis at an incidence rate of 102 (95% CI, 77.6 to 135) cases per 1,000 person-years. Overall, ≤ 10.1% of survivors had an emergency department or hospitalization visit. Oncologist services were overutilized, with a median of 6 (interquartile range: 4-10) visits in the first 6 months before reducing to a median of 2 (interquartile range: 1-3) visits biannually 3 years post-treatment. Bone mineral density test utilization rate adhered to guidelines for 92.2% of aromatase inhibitor recipients but only for 36.4% of premenopausal tamoxifen recipients.

**CONCLUSION:**

Overall, adherence rates to surveillance and osteoporosis preventive care were high. Extensive utilization of oncologist services up to 5 years post-primary treatment could be reversed with strategies to engage and coordinate survivorship care with primary care providers, leveraging their strengths to improve adherence to health promotion and chronic disease management.

## INTRODUCTION

Breast cancer is the most commonly diagnosed cancer among women worldwide, with an estimated 2.3 million new cases in 2020.^1^ In Singapore, the breast cancer incidence rate rose about 3.5 times between 1968-1972 and 2014-2018, from 20.1 to 70.7 per 100,000 population.^2^ Concurrently, early detection and the effective use of therapies have drastically improved the 5-year age-standardized relative survival rate to 81.4% in 2014-2018.^2^ This rising incidence, coupled with improved survival, contributed to a growing cohort of breast cancer survivors in the community. According to the National Cancer Institute, a person with a cancer diagnosis is termed as a survivor from the time of diagnosis to the end of life.^3^ After completing primary treatment, a survivor transits into an extended survivorship phase that usually encompasses 5 years of close surveillance for recurrence or secondary cancers.^4^ Long-term adverse events and symptoms such as cognitive changes, fatigue, and neuropathy may persist and continuously pose a challenge to survivors who are eager to re-establish normalcy in daily functioning.^5-7^ Survivors also experience psychosocial issues such as anxiety, body image concerns, and fear of recurrence.^8-12^ Furthermore, as chronic conditions such as cardiovascular diseases share similar risk factors as breast cancer, survivors may be predisposed to a higher comorbidity burden in the later survivorship phase.^8,13^

**CONTEXT**

**Key Objective**
In the absence of nationally endorsed practice guidelines, how adherent is current breast cancer survivorship care in Singapore to international follow-up care guidelines?
**Knowledge Generated**
We observed high adherence to guidelines in surveillance and osteoporosis preventive care for aromatase inhibitors recipients, but not for premenopausal tamoxifen recipients. Extensive utilization of oncologist services up to 5 years after primary treatment was also observed.
**Relevance**
Evaluation of guidelines adherence revealed existing care gaps and opportunities. Bone health should be managed appropriately according to guidelines regardless of menopausal status. To reduce the overutilization of oncologist services, primary care services could be increasingly engaged to share care roles with oncologists, especially in the later survivorship phase. National efforts to educate primary care providers on principles of cancer survivorship and relevant guidelines should be promoted, ensuring that surveillance mammograms would be offered after discharge from tertiary care.

To manage the different types of care needs among breast cancer survivors during survivorship, the Institute of Medicine (IOM) highlighted four core components of survivorship care in their landmark report: (1) prevention and detection of new cancers; (2) recurrent cancer surveillance; (3) interventions to address physical, psychosocial, and practical problems; and (4) well-coordinated care between specialists and primary care providers.^14^ Correspondingly, breast cancer survivorship care guidelines published by ASCO detailed recommendations to address the multifaceted survivorship problems.^15^

Currently, there is a lack of nationally endorsed practice guidelines for cancer survivorship care provision in Singapore. On the basis of a previous survey, Asian oncology practitioners reported that follow-up care focused on monitoring for physical and treatment-related adverse effects, with infrequent communication with nononcology health care practitioners.^16^ Although these self-reported findings suggest potential adherence to some aspects of the guidelines, the extent of compliance is unknown, given that survivors' comorbidity profile and health care utilization patterns after primary treatment are not well elucidated. A better understanding of survivorship care provided can help to identify care gaps and underutilized or overutilized services. Thus, this study aims to assess the quality of cancer survivorship care among breast cancer survivors in Singapore on the basis of the level of compliance with the ASCO guidelines, which addressed the four survivorship care components outlined by IOM. The characterization of breast cancer survivors' comorbidity burden, health care utilization patterns, and care practices serve to guide future strategies to improve care delivery.

## METHODS

### Study Cohort

This study analyzed a subgroup of breast cancer survivors from 217 participants who were recruited at the National Cancer Centre Singapore, Changi General Hospital, and KK Women's and Children's Hospital between November 2011 and September 2015. This study was granted ethical approval from the SingHealth Institutional Review Board (CIRB 2014/754/B) and conformed to Helsinki Declaration guidelines. The full cohort of recruited participants were (1) age ≥ 21 years, (2) diagnosed with nonmetastatic breast cancer, and (3) yet to undergo chemo/radiotherapy and scheduled to receive anthracycline- or taxane-based chemotherapy, with the primary aim to evaluate cognitive symptoms in breast cancer survivors from pretreatment to post-treatment. Thus, survivors diagnosed with neuropsychiatric or neurologic medical conditions were excluded. Informed consent was obtained from all participants. In this study, we analyzed survivors who (1) were 3 years or more out of primary treatment, and (2) did not experience breast cancer recurrence or develop secondary malignancies.

### Data Source

The public health care system in Singapore comprises three integrated health clusters.^17^ Each cluster includes tertiary hospitals, specialist outpatient clinics, community hospitals, community-based polyclinics, and partnering general practitioners. Detailed electronic medical records are shared within each cluster but not across. Participant recruitment sites belonged to the SingHealth cluster. Thus, electronic medical records from institutions within this cluster served as the primary data source for this study. Survivors' clinical notes and medical data were retrieved and reviewed.

### Data Collection

Data were collected between September 2018 and December 2018. A data collection form was created to collect demographic data such as age and race, cancer-related information on the breast cancer stage, and the types of treatment received. The start date for follow-up was defined as the day after the completion of primary active treatment (surgery, radiotherapy, and chemotherapy). Subsequently, each survivor's follow-up period was stratified into 6-month blocks. Survivors were followed up for a maximum of 5 years after primary active treatment or until December 2018, whichever occurred earlier.

### Outcomes

Adherence to the ASCO cancer survivorship care guidelines was assessed on the basis of the four survivorship care areas highlighted by IOM, as follows^14^:

1. Recurrent cancer surveillance: Adherence to surveillance was determined by the proportion of survivors with completed annual mammograms.
2. Monitoring and detecting late effects: We examined chronic diseases that share similar risk factors as cancer or are late effects of cancer and its treatment, including hyperlipidemia, type II diabetes, cardiovascular diseases, and osteoporosis.^18-20^ In this study, cardiovascular diseases included stroke, ischemic heart disease, arrhythmias, congestive heart failure, and hypertension. At baseline, the number and prevalence of chronic conditions were summarized. Thereafter, the incidence rate of each chronic disease diagnosed during follow-up was determined.
3. Health care resource utilization: ASCO guidelines recommended a follow-up frequency of every 3-6 months within the first 3 years and every 6-12 months in the subsequent 2 years for cancer survivorship issues. Thus, we assessed the number of visits to oncology disciplines (medical, surgical, or radiation) during follow-up. Exploratory analyses were conducted to identify demographic or clinical factors potentially associated with underutilization/overutilization. We further tabulated primary care visits to SingHealth polyclinics as an indicator of primary care services engagement. Finally, acute health care resource utilization was also determined by assessing the proportions of survivors who had one or more emergency department visit(s) or hospitalization episode(s).
4. Preventive care: According to national screening guidelines,^21^ adherence to second colorectal cancer screening was determined by the proportion of survivors age ≥ 50 years with a completed colonoscopy during the follow-up period. Second, adherence to regular bone mineral density (BMD) tests was assessed for the following subgroups: (1) aromatase inhibitors recipients and (2) premenopausal tamoxifen recipients. We defined premenopausal as age < 50 years according to the mean age in Singapore women.^22^ We characterized potential overuse of BMD tests for postmenopausal tamoxifen recipients. The proportion of survivors prescribed with recommended nutritional supplementation (calcium/vitamin D) for bone health was also tabulated. Data were further stratified by the osteoporosis status to reveal potential differences.

### Statistical Analysis

All data were analyzed using STATA version 17. We analyzed all available data up to 5 years after primary treatment. Descriptive statistics were used to summarize the baseline demographic and clinical characteristics and outcomes. Frequencies and percentages were used for categorical data. Means and standard deviations were used for normally distributed continuous data, whereas median and interquartile range (IQR) were used for continuous non-normally distributed data. The sample sizes across the follow-up period in 6-month intervals were specified, reflecting the number of participants with available data. Kaplan-Meier analysis was performed to determine the incidence rates of each chronic condition on the basis of recorded diagnoses. Univariate analyses were performed to identify factors possibly associated with the number of oncology visits before a multivariable negative binomial regression analysis was performed. Both Kaplan-Meier and regression analysis accounted for the time followed up, adjusting for missing data in later time points for some participants.

### Ethics and Other Permission

Ethics approval was obtained from the SingHealth Centralised Institutional Review Board (CIRB 2014/754/B).

## RESULTS

### Cohort Characteristics

Among 217 participants in the original cohort, a total of 189 survivors satisfied the inclusion criteria and were analyzed (Table 1). The mean age of survivors was 56.9 (SD 8.6) years. Data from the majority (139/189, 73.5%) of the survivors were available beyond 3 years after primary treatment, with approximately one quarter (47/189, 24.9%) available for up to 5 years after primary treatment. The majority were Chinese (154/189, 81.5%) and were diagnosed with stage I/II breast cancer (150/189, 79.4%). Most survivors underwent surgery (184/189, 97.4%) and chemotherapy (183/189, 96.8%). Approximately half (93/189, 49.2%) of the survivors did not have any chronic conditions investigated at baseline. Most survivors were initiated on tamoxifen (53/81, 65.4%) than an aromatase inhibitor (28/81, 34.6%) for endocrine therapy.

**TABLE 1:**
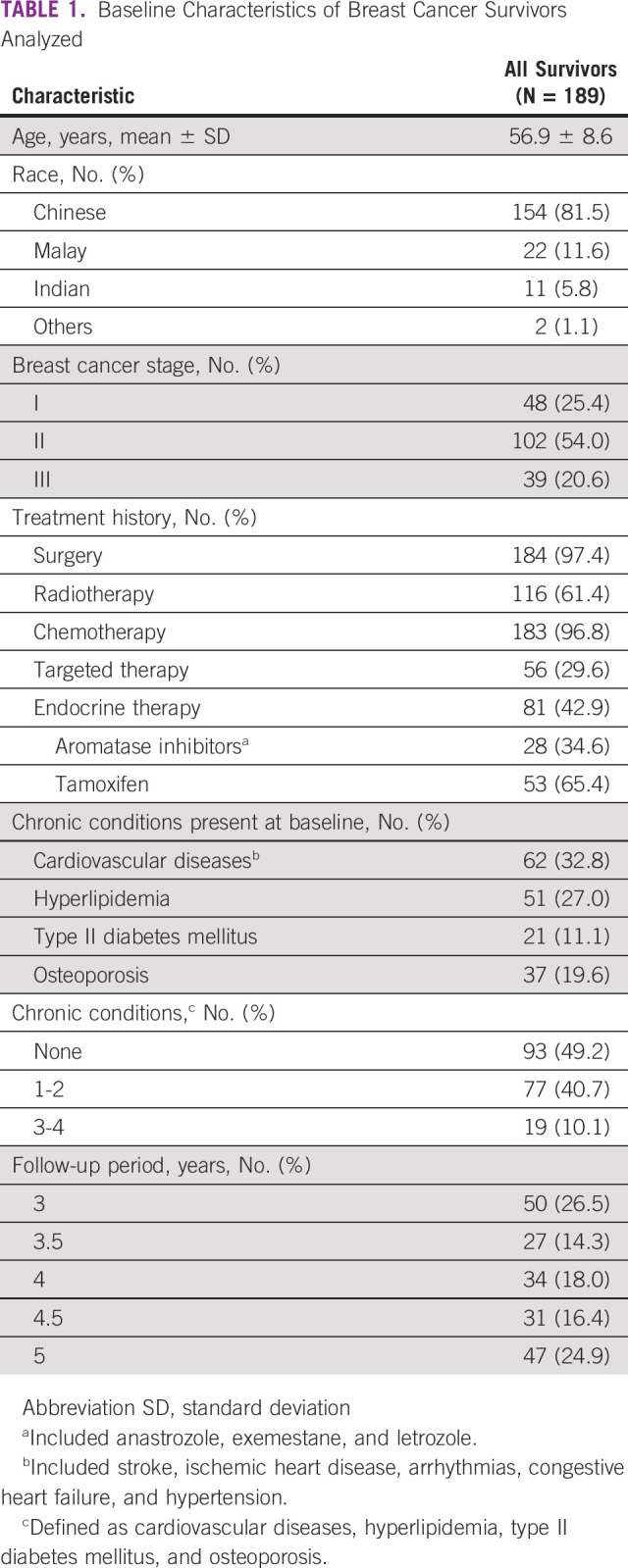
Baseline Characteristics of Breast Cancer Survivors Analyzed

#### Recurrent cancer surveillance.

Adherence to annual surveillance mammography was consistently ≥ 83% over the follow-up period with overall low overutilization of mammograms (Table 2).

**TABLE 2:**
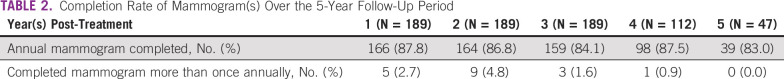
Completion Rate of Mammogram(s) Over the 5-Year Follow-Up Period

#### Monitoring and detecting late effects.

**FIG 1:**
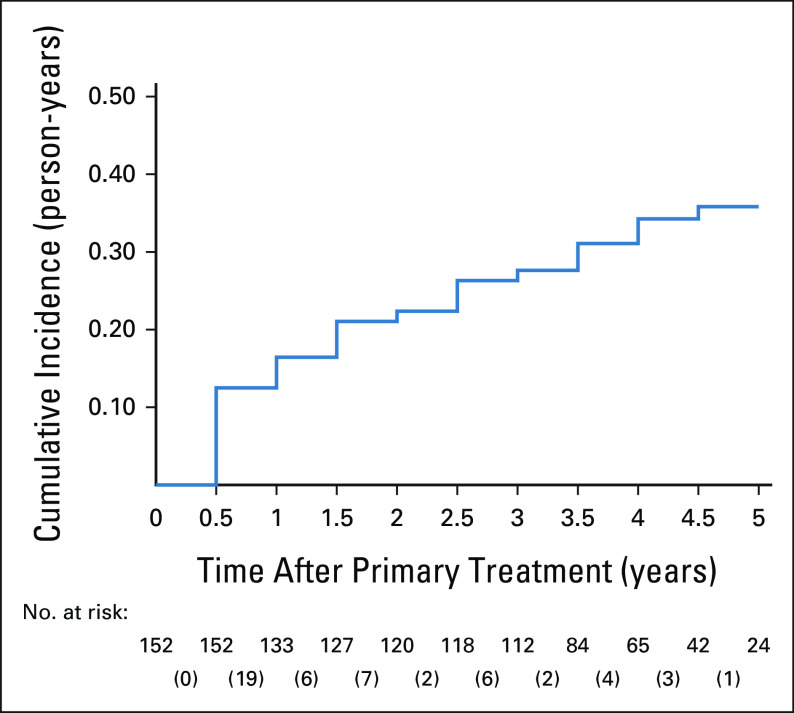
Cumulative incidence of osteoporosis among breast cancer survivors without osteoporosis diagnosis at initial point of follow-up with risk table at 6-month intervals. The number of incident cases at each 6-month interval is shown in the corresponding bracket.

The baseline prevalence of chronic diseases in decreasing order is as follows: 62/189 (32.8%) had cardiovascular diseases, 51/189 (27.0%) had hyperlipidemia, 37/189 (19.6%) had osteoporosis, and 21/189 (11.1%) had diabetes (Table 1). The most common new diagnosis documented was osteoporosis, with an incidence rate of 102 (95% CI, 77.6 to 135) cases per 1,000 person-years (Table 3). Figure 1 shows the cumulative incidence curve for osteoporosis, with a steep increase in new diagnoses in the first 6 months. The change in the comorbidity burden over time reflected the new diagnoses and is summarized in Appendix Table A1.

**TABLE 3:**
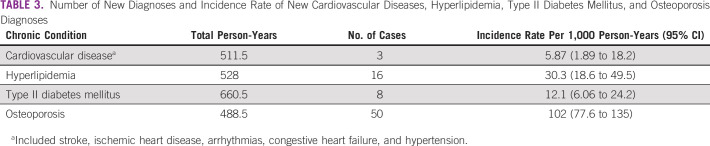
Number of New Diagnoses and Incidence Rate of New Cardiovascular Diseases, Hyperlipidemia, Type II Diabetes Mellitus, and Osteoporosis Diagnoses

#### Health care resource utilization.

The number of consultations with oncologists in 6-month intervals reduced from 6 (IQR: 4-10) in the first 6 months post-treatment to 2 (IQR: 1-3) 3 years post-treatment, before stabilizing (Table 4). These visit frequencies reflected overutilization of oncologist services on the basis of guideline recommendations. Further univariate analyses identified stage III cancer, treatment history of radiotherapy, and targeted therapy as potential factors associated with high oncologists' services utilization (Appendix Table A2). In the multivariable analysis, survivors with a history of targeted therapy had a 1.47 (95% CI, 1.32 to 1.63) times higher rate of oncologist visits than survivors not treated with targeted therapy, after adjusting for cancer staging and radiotherapy treatment history. For nononcologist visits, less than half of the cohort had consultations in the community in the first 2 years after primary treatment, after which, the utilization of such primary care resources gradually increased to involve > 50% of the cohort (Table 4). Overall, ≤ 10.1% of survivors used acute resources during follow-up. The highest utilization was observed within the first 6 months with 10.1% (19/189) and 9.5% (18/189) of survivors with at least one emergency department visit and hospitalization, respectively.

**TABLE 4:**
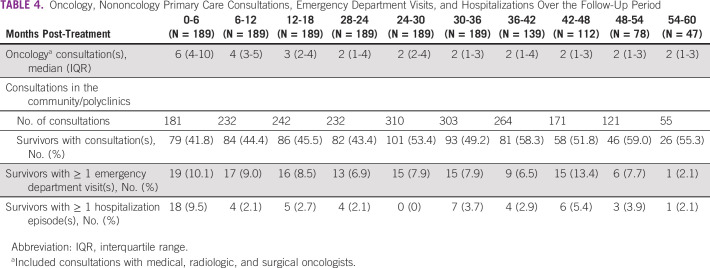
Oncology, Nononcology Primary Care Consultations, Emergency Department Visits, and Hospitalizations Over the Follow-Up Period

#### Preventive care.

For colorectal cancer screening, 14 of 150 (9.3%) survivors age ≥ 50 years had a colonoscopy record. Among survivors receiving endocrine therapy, 69 and 81 survivors were initiated on aromatase inhibitors and tamoxifen, respectively, either at baseline or during follow-up. A total of 32 survivors experienced a switch in therapy, where 11/69 (15.9%) switched from aromatase inhibitors to tamoxifen, and 21/81 (25.9%) switched from tamoxifen to aromatase inhibitors.

The BMD tests performed and nutritional supplementation prescribed during follow-up are summarized in Table 5. Among 90 aromatase inhibitors recipients, 92.2% had one or more BMD test(s), with a median number of 2 (IQR: 1-3) tests documented. The majority (87/90, 96.7%) received nutritional supplementation, consistent with guidelines' recommendations for this high-risk group. For premenopausal tamoxifen recipients, the minority (12/33, 36.4%) received one or more BMD test(s) despite being identified as a high-risk group in the guidelines. Most survivors who received tests were also diagnosed with osteoporosis during follow-up (7/12, 58.3%). Despite a lower proportion than aromatase inhibitors recipients, the majority (23/33, 69.7%) received nutritional supplementation. Finally, possible overuse of BMD tests was observed for 36/59 (61.0%) postmenopausal tamoxifen recipients, with an overall high supplementation coverage at 88.1% (52/59).

**TABLE 5:**
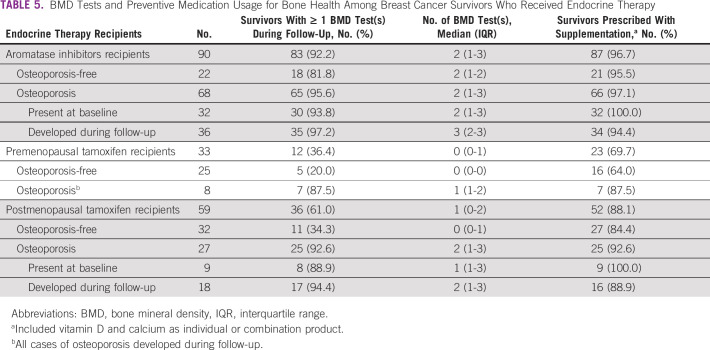
BMD Tests and Preventive Medication Usage for Bone Health Among Breast Cancer Survivors Who Received Endocrine Therapy

## DISCUSSION

We assessed the quality of cancer survivorship care among nonmetastatic breast cancer survivors in Singapore in four areas: recurrent cancer surveillance, monitoring and detecting late effects of cancer and treatment, health care resource utilization, and preventive care. Overall, adherence rates to surveillance and osteoporosis preventive care were high. We observed a generally low comorbidity burden in this cohort on the basis of documented diagnoses. Acute health care resource utilization was low during survivorship, whereas oncologists' services were overutilized when compared with guideline recommendations.

Our assessment revealed strengths and areas for improvement in current survivorship care provision. High adherence to annual mammograms of ≥ 83% up to 5 years after primary treatment reinforced the current survivorship care focus on surveillance.^23^ Evaluations among similar breast cancer cohorts in other high-income countries such as the United States and the Netherlands revealed similarly high adherence rates > 80% up to the fifth survivorship year.^24,25^ Although not assessed in the current study, long-term adherence beyond the fifth year would be of interest as the US study showed a decreasing trend from the fifth year onward.^24^ Survivors discharged from tertiary settings in late survivorship may have suboptimal adherence to surveillance mammograms in the primary care setting, highlighting a potential care gap. In contrast to surveillance, colorectal cancer screening appeared to be less common on the basis of the number of colonoscopies performed. However, the true screening rate was likely underestimated by our methodology because of inadequate follow-up time, as the recommended interval of 5-10 years exceeded the maximum follow-up duration.^26^ Furthermore, alternative subsidized screening strategies such as fecal immunochemical tests are routinely performed in primary care sites through the National Colorectal Cancer Screening Program.^26^ We were unable to capture these data to allow a conclusive assessment of colorectal cancer screening compliance.

For osteoporosis preventive care, the BMD test utilization rate among aromatase inhibitors recipients adhered to guidelines, faring better than an Israeli cohort of comparable age where a smaller proportion of survivors with at least one test was observed (73.5% *v* 92.2%).^27^ Complemented with high coverage of nutritional supplementation, practitioners demonstrated high awareness of this high-risk group. On the contrary, suboptimal preventive screening was observed among premenopausal tamoxifen recipients. Premenopausal survivors who are typically younger (< 50 years) and without typical risk factors for osteoporosis may be overlooked by practitioners when initiated on tamoxifen. Moreover, although regular testing of postmenopausal tamoxifen recipients was not routinely recommended, the overall high testing rate in this group could be attributed to other risk factors such as age, potentially also explaining the high coverage of nutritional supplementation (88.1%) provided.

Monitoring and detecting late effects of breast cancer is pertinent to manage survivors' health holistically beyond treatment. The incidence rates of chronic diseases in our cohort were in the same order of magnitude as the literature.^28-30^ However, large confidence intervals precluded precise estimation of incident disease detection rates. Notably, the incidence of osteoporosis at 102 per 1,000 person-years almost tripled the rate obtained in an Israeli study with a similar distribution of survivors receiving endocrine therapy.^27^ Routine BMD tests conducted before aromatase inhibitors initiation^15^ could have detected previously undiagnosed prevalent cases instead of true incident cases, driving a sharp increase in diagnoses in the first 6 months. Besides surveillance bias, the low comorbidity burden observed should be interpreted with caution. The proportion of survivors free from examined chronic conditions at 3 years post-treatment was 36.0%, higher than the 26.2% reported in another US study of a similar cohort.^31^ The deviation could arise from differences in data completeness and collection strategy, where the US study relied on self-reported comorbidity data. Our study likely underestimated the comorbidity burden because of missed diagnoses in institutions beyond our health cluster. Nevertheless, cancer survivors had a higher disease burden, evident from a higher prevalence of cardiovascular diseases and diabetes than the national population,^32^ emphasizing the importance of corresponding management during follow-up.

Follow-up consultations with oncologists reduced to a median frequency of four times yearly at 5 years after primary treatment, doubling the recommended frequency. Incoherence with recommended follow-up frequency suggests potential overuse of tertiary resources in the late survivorship phase. This overutilization trend was also reported in other studies, albeit at a lower frequency in a Dutch study and in a Canadian study where > 50% of survivors received more visits than recommended.^25,33^ This trend warrants concern as a systematic review showed that more intensive follow-up did not confer additional mortality benefits over less intensive follow-up.^34^ Further analyses revealed targeted therapy treatment as a significant factor associated with extensive oncologist visits. Associated increase in visits could be related to cardiac-related monitoring for survivors who received trastuzumab. A lack of consensus over the optimal surveillance frequency and modality potentially resulted in practice variations.^35^ Without other significant demographic and clinical factors, the traditional perception of cancer in Singapore as a disease to be exclusively managed in the tertiary health care settings may have contributed to this trend.^23^ Furthermore, overutilization of oncology services may incur opportunity costs with possible underutilization of primary care, leading to suboptimal management of other cancer survivorship issues such as comorbidities and preventive health support.^36-38^ Systematic reviews have shown that involvement of primary care in survivorship care is safe, leading to similar clinical outcomes as tertiary care with potential cost-savings.^39,40^ Encouragingly, we also observed an increasing proportion of survivors with primary care consultations during follow-up, highlighting opportunities for primary care providers to play a more active role in cancer survivorship provision.

To our knowledge, this is the first study to evaluate breast cancer survivorship care provision in Singapore holistically by benchmarking practices against international survivorship care guidelines. Our study has some limitations. Foremost, medical records at private general practitioners' clinics and institutions beyond the health cluster were inaccessible. As private providers constitute a significant proportion of primary care, we likely underestimated primary care visits and new diagnoses in the community.^41^ Furthermore, data accuracy and reliability were not validated in this study, potentially contributing to underestimation of comorbidity burden. Data availability also limited the scope of our analysis. Other late effects such as anxiety and depression, as well as risk profiles such as smoking status, alcohol consumption, and physical activity, were not systematically available for retrieval. This study was not able to explore whether extensive oncologist visits reflected risk-stratified personalized care. Finally, excluding survivors with recurrences in this study means that results on care quality were only representative of a cancer-free survivor cohort and should not be extended to survivors with secondary malignancies.

Our results revealed a practice gap in monitoring for bone health among premenopausal survivors initiated on tamoxifen, highlighting a need to improve recognition of this susceptible group. Given the oncologist-centric focus in Singapore, it may not be feasible to conform to oncologist visit frequencies recommended by guidelines. Instead, strategies could be used to reverse the overutilization of oncologist services. First, evidence-based findings that more intensive follow-up care does not confer additional survival benefits should be communicated to survivors, assuring survivors who lacked security in fear of worse outcomes. Second, increasing engagement with primary care services throughout survivorship may present opportunities for cancer survivorship care delivery in the community. National efforts to educate primary care providers on principles of cancer survivorship and relevant guidelines should be promoted, ensuring that surveillance mammograms would be offered after discharge from tertiary care. Future work could explore shifts in care roles from specialist centers to the community, especially in the later survivorship phase, by leveraging primary care strengths.

In conclusion, most nonmetastatic breast cancer survivors in Singapore had at least one chronic condition requiring management during their survivorship phase, with comorbidity burden likely underestimated in this study. Although current cancer survivorship care provision in Singapore is adherent to international guidelines in surveillance and osteoporosis preventive care, our results highlighted an extensive utilization of oncologist services up to 5 years after primary treatment. Concurrently, increased utilization of primary services in late survivorship suggests opportunities to engage and coordinate care with primary care providers. Their involvement would complement current oncologists' strengths and improve adherence to other aspects of the guidelines, including health promotion and chronic disease management.

## Figures and Tables

**TABLE A1:**
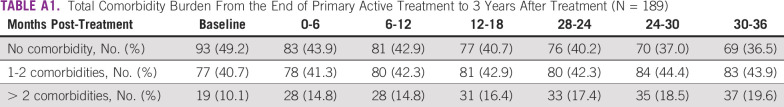
Total Comorbidity Burden From the End of Primary Active Treatment to 3 Years After Treatment (N = 189)

**TABLE A2:**
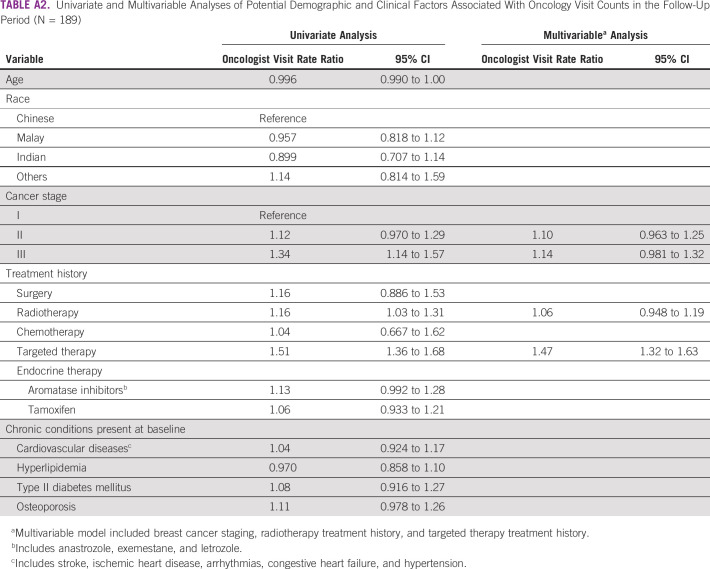
Univariate and Multivariable Analyses of Potential Demographic and Clinical Factors Associated With Oncology Visit Counts in the Follow-Up Period (N = 189)

## Data Availability

The data underlying this article will be shared on reasonable request to the corresponding author.
